# Methotrexate-induced pneumonitis in a patient of rheumatoid arthritis

**DOI:** 10.11604/pamj.2025.50.13.44591

**Published:** 2025-01-07

**Authors:** Sumer Choudhary, Gaurang Aurangabadkar

**Affiliations:** 1 Department of Respiratory Medicine, Datta Meghe Medical College, Nagpur, India,; 2 Datta Meghe Institute of Higher Education and Research, Deemed University, Sawangi, Wardha, Maharashtra, India

**Keywords:** Rheumatoid arthritis, methotrexate, pneumonitis

## Image in medicine

A 58-year-old male patient presented with chief complaints of pain and deformities in small joints of both hands and feet along with non-productive cough and dyspnea on exertion since 3 months. The patient gave a history of being diagnosed as a case of rheumatoid arthritis 6 months back and was started on methotrexate therapy by the rheumatologist, as per protocol, after doing screening Computerized Tomography (CT) thorax prior to starting therapy which was demonstrated to be normal (A). The patient was advised regular follow-up testing for liver function tests and Chest X-ray after 2 months of starting methotrexate therapy. However, the patient was lost to follow-up and did not undergo the same as per schedule. The CT scan of the chest was done (B) which revealed the presence of ground-glassing and interlobular septal thickening in the left lung and overlapping nodular opacities with sub-pleural opacities in the right lung. The patient was radiologically diagnosed as a case of pulmonary tuberculosis and was started on anti-tubercular therapy by the general practitioner. However, in spite of 2 months of anti-tubercular therapy, the patient failed to show improvement. Bronchoscopy of the patient was done and the Broncho-Alveolar Lavage (BAL) GeneXpert testing was negative for *Mycobacterium tuberculosis* and culture showed no growth of any identifiable organism. A decision was taken to stop the methotrexate therapy for the patient and the patient was observed for 15 days after which the patient began to demonstrate gradual clinical and radiological improvement. Therefore, a diagnosis of methotrexate-induced pneumonitis was made based on the investigations and the history of the patient. Methotrexate is a first line drug used in the management of rheumatoid arthritis. It is however associated with a 2-7% risk of developing adverse pulmonary effects, with the most common finding being an acute hypersensitivity pneumonitis.

**Figure 1:**
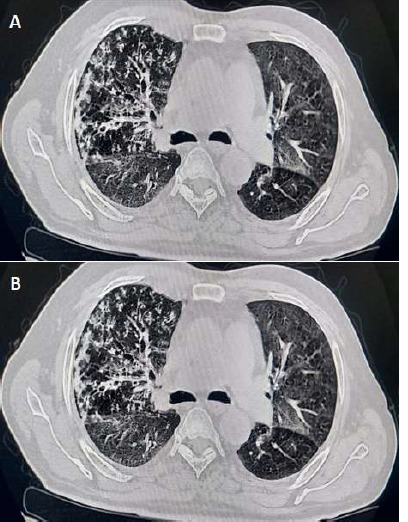
A) computerized tomography of the thorax prior to starting methotrexate therapy; B) high-resolution computed tomography scan of the thorax showing ground-glass opacities and interlobular septal thickening in the left lung and overlapping nodular and sub-pleural opacities in the right lung

